# Comparative Evaluation of the Antibacterial Efficacy of Metronidazole, Chlorhexidine, and Normal Saline as Laser- and Sonic-Activated Intracanal Irrigants Against Enterococcus faecalis: In Vitro Study Protocol

**DOI:** 10.2196/76783

**Published:** 2026-01-07

**Authors:** Sharvari Deshmukh, Aditya Patel

**Affiliations:** 1 Department of Conservative Dentistry and Endodontics, Sharad Pawar Dental College & Hospital, DMIHER Campus, Sawangi-Meghe, Wardha, India, 91 9373773020

**Keywords:** *Enterococcus faecalis*, metronidazole, chlorhexidine, sonic activation, laser activation, root canal disinfection, intracanal irrigants, endodontic treatment

## Abstract

**Background:**

The persistence of *Enterococcus faecalis* is a significant challenge in endodontic therapy, due to its ability to form biofilms and penetrate the dentinal tubules, frequently leading to treatment failures. Therefore, despite the proven antimicrobial activity of metronidazole (MTR) and chlorhexidine (CHX), the resilience of *E. faecalis* necessitates integrating activation techniques. Laser and sonic activation methods show promise in enhancing the antibacterial performance of irrigants. However, studies on the activation-enhanced efficacy of MTR, CHX, and saline remain limited.

**Objective:**

This study aimed to compare the antibacterial efficacy of MTR, CHX, and normal saline as intracanal irrigants when activated by laser and sonic techniques against *E. faecalis* in extracted human mandibular premolars.

**Methods:**

It is a laboratory-based in vitro experimental study involving extracted human teeth. Ninety freshly extracted single-rooted mandibular premolars will be obtained, decoronated, and biomechanically prepared. Canals will be inoculated with *E. faecalis* (ATCC 29212) and incubated for 7 days to allow biofilm formation. The teeth will then be randomly divided into 3 main groups ([MTR], [CHX], and saline), each subdivided based on the activation method used (laser or sonic). Irrigation protocols will be standardized, and pre- and postirrigation bacterial samples will be collected on paper points and cultured on brain heart infusion agar. Colony-forming units will be counted to evaluate antibacterial efficacy.

**Results:**

As of June 2025, ethical approval for the study has been obtained. Sample collection of extracted human teeth commenced in July 2025, and 90 extracted teeth have been collected as of January 2026, corresponding to the planned sample size. Laboratory procedures and data analysis have not yet commenced.

**Conclusions:**

This study aims to provide evidence-based insights into optimizing root canal disinfection protocols by evaluating and comparing the synergistic effects of antimicrobial agents and advanced activation techniques. The findings could contribute significantly to improving endodontic treatment outcomes and reducing the incidence of persistent infections caused by *E. faecalis*.

## Introduction

The combined action of intracanal drugs and chemomechanical preparation reduces or eliminates bacteria during root canal therapy (RCT) [1]. The mechanical action of tools and the flow and backflow of irrigating solutions are used to remove bacteria and other irritants from the root canal’s boundaries [2]. A common and successful endodontic treatment for pulpitis and apical periodontitis is conventional root canal therapy, which uses biomechanical equipment to remove affected tooth pulp and dentin tissue while using antibiotics and antibacterial materials to eradicate germs [3].

*Enterococcus faecalis *has been detected in approximately 4%-40% of primary endodontic infections, indicating its significant role in persistent and recurrent root canal infections, especially in cases of treatment-resistant apical periodontitis [4,5]. Despite root canal treatment, up to 90% of teeth may retain residual infection. In the twenty-first century, with rising advancements in RCT, the complete eradication of *E. faecalis* remains challenging due to its ability to survive under adverse conditions, with 2 main pillars of its survival being penetrating dentinal tubules and forming biofilms that resist conventional antimicrobial strategies. Its biofilms are notably resilient to commonly used intracanal agents like sodium hypochlorite and calcium hydroxide [6].

Many medications, including metronidazole (MTR) and chlorhexidine (CHX), have been used to treat oral diseases because they are excellent at inhibiting bacteria and biofilms. A broad-spectrum antibiotic that targets both anaerobic bacteria and protozoa, MTR is a nitroimidazole molecule. In addition to Gram-positive and Gram-negative bacilli, it exhibits strong antibacterial activity against anaerobic cocci. Both topical and systemic applications have been made in the treatment of periodontal disease. The anatomical complexity of the RCT may, however, harbor germs and remaining pulp tissue following RCT, potentially leading to treatment failure [7].

CHX is a cationic biguanide with broad-spectrum coverage. It has activity against both Gram-positive and Gram-negative bacteria. At low concentrations with a range between 0.2% and 0.06%, CHX shows bacteriostatic properties. At high concentrations, more than 0.12%, it depicts bactericidal activity, antifungal properties, and some antiviral properties. Its effect is not hampered by biological fluids such as blood, and it works for up to 48 hours after application. It acts by penetrating the bacterial cell membrane, causing cytoplasmic leakage at lower concentrations and precipitating nucleic acid at higher concentrations [8].

Normal saline, although lacking intrinsic antimicrobial properties, is included in this study as a negative control to isolate and evaluate the specific contribution of activation techniques (laser and sonic) independent of chemical disinfection. Its inclusion enables assessment of whether activation alone can produce any measurable reduction in bacterial load.

The distribution method, in conjunction with the irrigant’s characteristics, affects the irrigation efficacy [9]. Irrigant activation in endodontic procedures pertains to the use of various energy forms—mechanical, sonic, ultrasonic, or other physical modalities—to enhance the dynamic movement and distribution of irrigating solutions throughout the intricately structured root canal system. Evidence from systematic reviews and meta-analyses indicates that the use of activation, regardless of the specific device or mechanism employed, significantly improves the efficacy of debris and smear layer removal. Consequently, activation is recognized as an integral phase in the chemo-mechanical debridement process, contributing to the overall success of root canal disinfection [10]. In contrast to ultrasonic irrigation, sonic irrigation uses a lower frequency (1‐10 kHz). As a result, shear wall stresses and fluid velocity are reduced. Conversely, sonic activation produces noticeably more amplitude (horizontal tip displacement) [11].

In recent years, nonsurgical RCT has made greater use of laser technology. When used in conjunction with chemical solutions, its high power and distinct coherent rays are helpful during the root canal cleaning and disinfection phase. It has been demonstrated that a laser can effectively activate a root canal irrigant by causing mechanical disruptions [12]. The 940 nm diode laser is frequently used in dentistry clinics due to its compact size, portability, affordability, and broad variety of oral medicine applications. Bacterial cell walls can be destroyed by the bactericidal photothermal disrupting impact of its radiation [13]. Since water and hydroxyapatite are the primary components of dentin, the 940 nm wavelength has low absorption coefficients in water (about μa=0.04‐0.05 cm^−1^), which allows the laser beam to penetrate deeply into dentin (over 1000 μm) and kill distant bacteria [13,14].

It has been observed in previous studies that the use of CHX and MTR as intracanal irrigants showed optimal results. However, studies with MTR as an intracanal irrigant with different activation methods need to be explored. Although metronidazole has demonstrated antimicrobial efficacy as an intracanal irrigant, prior studies have primarily evaluated it in static or nonactivated forms. So, there is a need for research that compares the antibacterial effectiveness of MTR, CHX, and normal saline as intracanal irrigants, which are activated by laser and sonic activation methods, against *E. faecalis*. This comparison will help determine the most effective activation technique for enhancing the antibacterial effectiveness of these irrigants, ultimately contributing to more reliable and durable endodontic treatments, improving treatment success rates, and reducing the incidence of persistent infections associated with this resilient bacterium. This study might address this critical gap by evaluating the synergistic effects of activation-enhanced MTR, thereby expanding current understanding and potentially informing more effective endodontic disinfection protocols.

This in vitro study aims to compare the antibacterial efficacy of MTR, CHX, and normal saline as intracanal irrigants when activated using laser and sonic techniques against *E. faecalis* in extracted human mandibular premolars. The study evaluates the effectiveness of each irrigant with both laser and sonic activation techniques to determine their potential in eliminating bacterial contamination from the root canal system. It is hypothesized that laser and sonic activation will significantly enhance the antibacterial efficacy of these irrigants, with notable differences among the groups.

The null hypothesis for the study assumes that, “there will be no significant effect of laser and sonic activation on the antibacterial efficacy of MTR, CHX, and saline as intracanal irrigants in eradicating Enterococcus faecalis in root canal treatments” while the alternative hypothesis suggests, “laser and sonic activation will significantly enhance the antibacterial efficacy of MTR, CHX, and saline as intracanal irrigants in eradicating Enterococcus faecalis in root canal treatments.”

## Methods

### Study Design

This study used a comparative in vitro study design.

### Materials

The materials to be used in the current study are listed in Textbox 1.

Textbox 1. **Materials to be used for microbial inoculation, irrigation, activation, and root canal preparation.**

- Microbiological sample preparation:

 1. Strains of *Enterococcus faecalis*
 2. Brain heart infusion (BHI) agar media
 3. Incubator

- Irrigants:

 1. Chlorhexidine (CHX)
 2. Metronidazole (MTR)
 3. Normal saline

- Activation devices:

 1. Sonic activation device
 2. Diode laser

- For sample preparation:

 1. Protaper Universal treatment rotary files (for biomechanical preparation)
 2. Paper points

### Sample Size

The sample size was estimated based on previously published data [15], assuming a power of 80%, significance level of 5%, and a mean difference of 23 in colony-forming unit (CFU) between groups. Group SDs were 17.33 and 2.81. These assumptions resulted in a minimum of 15 samples per subgroup. The complete sample size formula, derivation, and calculation steps are provided in Multimedia Appendix 1. Although the reference study involved a different irrigant (BioPure MTAD) and microbial model, it was the most comparable source available and served as a reasonable baseline for estimating variability in CFU reduction in the current study.

### Sample Selection

The inclusion and exclusion criteria are given in Textbox 2.

Textbox 2. **Inclusion and exclusion criteria.**

Inclusion Criteria

- Freshly extracted single-rooted permanent mandibular lower premolar teeth with closed apex, free from caries, fracture, and previous endodontic treatment.

Exclusion Criteria

- Teeth with visible cracks, abnormal morphology.
- Teeth with developmental anomalies.
- Teeth with external and internal resorption.
- Teeth with multiple roots.
- Teeth with calcified canals.

### Sample Allocation

Distribution of sample size was 90. The specimens will be randomly divided into 3 groups and 6 subgroups (Table 1).

**Table 1.** Sample distribution across experimental groups based on irrigant type and activation methodology.

Groups and subgroups	Samples (n)
Group I: 5 ml of 0.5% MTR^a^ as an irrigant for 3 min	
Subgroup IA: Laser activation for 30 sec	15
Subgroup IB: Sonic activation	15
Group II: 5 ml of 2% CHX^b^ as an irrigant for 3 min	
Subgroup IIA: Laser activation	15
Subgroup IIB: Sonic activation	15
Group III: 5 ml of normal saline as an irrigant for 3 min	
Subgroup IIIA: Laser activation	15
Subgroup IIIB: Sonic activation	15

a MTR: metronidazole.

b CHX: chlorhexidine.

### Groups

This study aims to evaluate the antibacterial efficacy of 3 irrigant solutions—MTR, CHX, and normal saline—against *E. faecalis* using 2 activation methods (laser and sonic). The study will involve a total sample size of 90, divided into 3 main groups based on the intracanal irrigants used, and will be further divided into subgroups based on the activation protocol employed.

### Sample Preparation

90 extracted single-rooted premolar teeth will be obtained from healthy patients with informed consent. Extractions will be performed for reasons unrelated to dental pathology, such as orthodontic treatment, prosthetic rehabilitation (eg, to facilitate fixed or removable partial dentures), esthetic concerns, or to preserve adjacent vital structures. Teeth exhibiting caries, restorations, root resorption, calcifications, or a history of endodontic treatment will be excluded from the study. In case of sample loss due to contamination, fracture, or invalid measurements during any phase of the study, replacements will be made from the remaining pool of eligible specimens to maintain balanced group sizes. All such instances will be recorded and reported.

### Tooth Decoronation and Canal Preparation

The teeth will then be cleaned and sterilized, removing any residual tissue. Following the removal of any remaining tissue, the teeth will be washed and sterilized before being kept at room temperature in a 0.1% thymol solution until the experimental phase. To achieve a uniform root length of 13 mm, the crowns will thereafter be decoronated using a diamond disc. To get the working length, 1 mm will be subtracted from the total root length. From the total root length to establish the working length, 1 mm will be deducted. Root canal shaping will be carried out up to the F2 instrument.

### Inoculation of *E. faecalis*

The strain of *E. faecalis* (ATCC 29212) used in this study will be obtained from the Department of Microbiology, Jawaharlal Nehru Medical College, Datta Meghe Institute of Medical Sciences (Deemed to be University), Wardha, India. The strains will be cultured and inoculated into the prepared canals of all samples. The samples will be incubated for 7 days to allow biofilm formation. Before irrigation, sterile paper points will be inserted into each canal for 60 seconds to collect pretreatment samples. These paper points will then be transferred to BHI Agar media and incubated for 48 hours. The colonies will then be counted using the classic bacterial counting method. Preirrigation samples will be taken. The inoculated teeth will then be randomly assigned to their respective groups and subgroups.

### Biosafety Measures

All procedures involving *E. faecalis* will be conducted in a biosafety level-2 laboratory, following institutional biosafety guidelines. Sterile gloves, masks, and lab coats will be worn during microbial handling. All contaminated instruments and waste will be autoclaved and discarded in biohazard disposal systems. Teeth will be stored in 0.1% thymol prior to use to maintain sterility.

### Irrigation Protocol

Irrigation will involve 3% sodium hypochlorite (Shivam, India), followed by 3 ml of 17% ethylene diamine tetra acetic acid for 1 minute to remove the smear layer, and subsequent irrigation protocol of every group will then be followed by using 5 ml of respective irrigants of 0.5% MTR, 2% CHX, and saline for 3 minutes accompanied by laser sonic activation with the EndoActivator (Dentsply Maillefer, Switzerland) operating at 10,000 cycles per minute (167 Hz) using a medium-sized tip (25/04). Each canal will be agitated for 30 seconds continuously. Laser activation will be performed using a diode laser (Biolase Epic X, Biolase Inc., USA) operating at a wavelength of 980 nm and a power setting of 1.5 W in pulsed mode. A 200-µm flexible fiber tip will be introduced 1 mm short of the working length. Each canal will undergo 3 cycles of laser activation, with each cycle lasting 5 seconds, followed by a 10-second interval between cycles. Postirrigation samples will be collected with the help of paper points and deposited into BHI Agar media and incubated for 48 hours.

### Outcome Measures

The colonies will then be counted using the classic bacterial counting method to evaluate bacterial reduction. The data collected will then be subjected to statistical analysis. Scanning electron microscopy can offer visual confirmation of biofilm formation. The present study will follow a validated 7-day *E. faecalis* incubation protocol, as established in previous literature. In phase I of the study, antimicrobial efficacy will be quantitatively assessed using CFU counts, which provide a reliable and clinically relevant measure.

**Figure 1.**
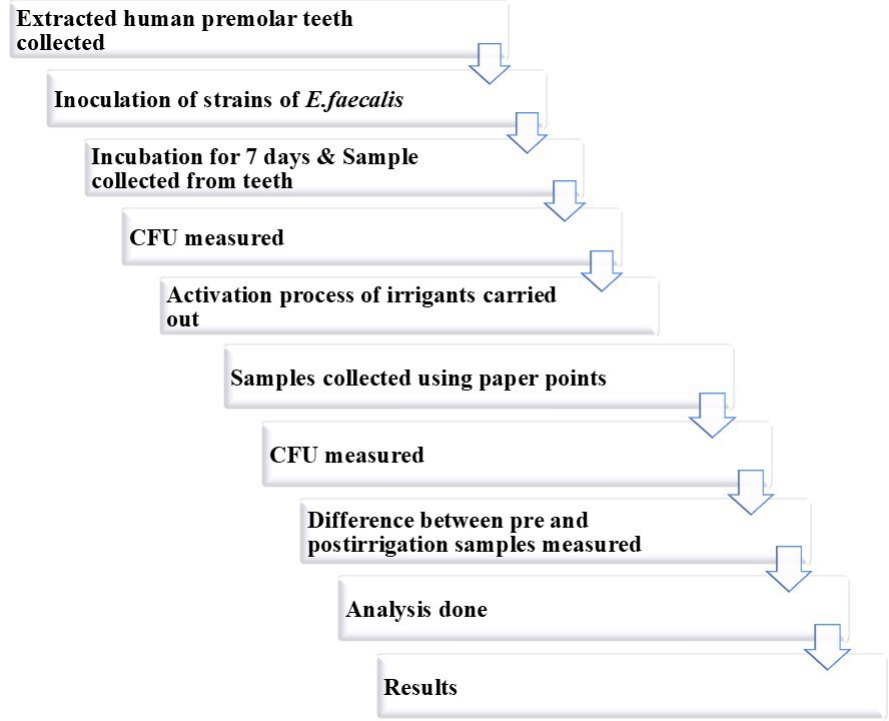
Experimental protocol workflow. CFU: colony-forming units; *E. faecalis*: *Enterococcus faecalis*.

The procedure to be followed is depicted in the following flowchart (Figure 1).

### Statistical Analysis

**Figure 2.**
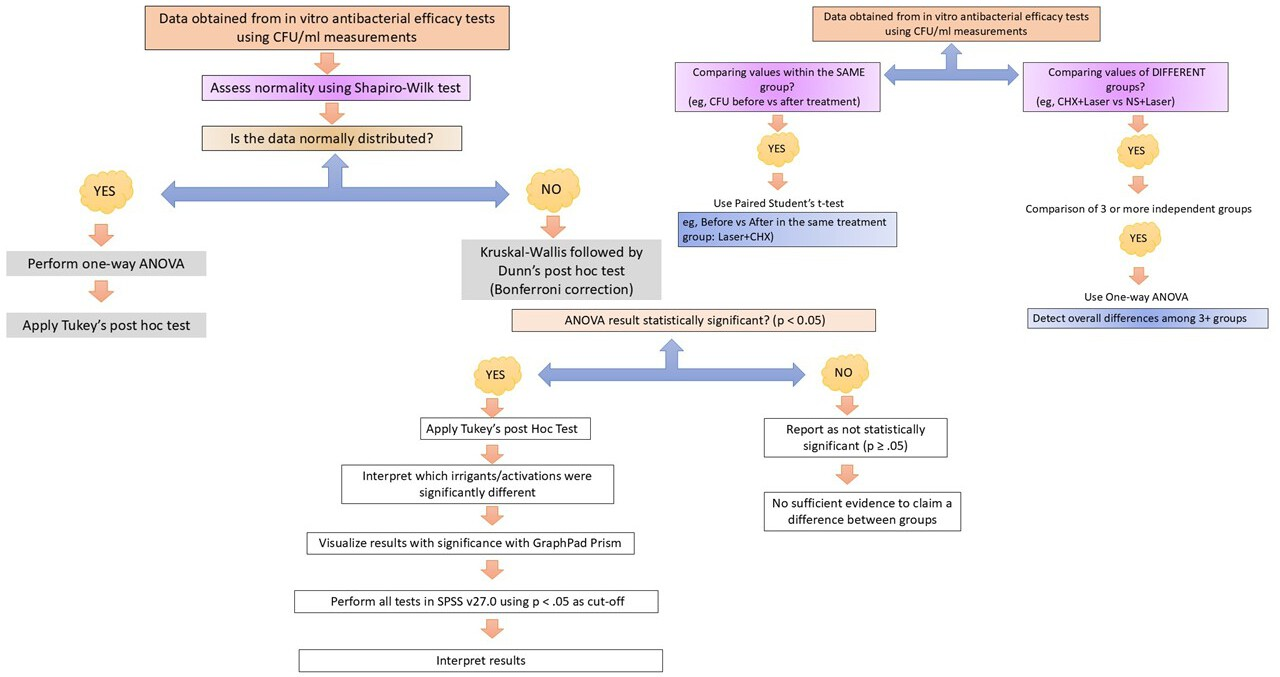
Stepwise statistical analysis workflow. CFU: colony-forming units; CHX: chlorhexidine; NS: normal saline.

The dataset will undergo both summarization and advanced statistical interpretation. Normality of data distribution will be assessed using the Shapiro-Wilk test. Analytical tools will include the 𝜒^2^ method, Student’s *t* tests (applicable for both related and independent samples), as well as one-way ANOVA followed by Tukey’s postanalysis comparisons. All statistical procedures and visual data representations will be executed using SPSS (version 27.0) and GraphPad Prism (version 7.0). A probability value *P*<.05 will be treated as indicative of a statistically meaningful result. The statistical methods to be applied are illustrated in Figure 2.

### Ethical Considerations

Ethical approval for the study was obtained from the Institutional Ethical Committee of Datta Meghe Institute of Higher Education & Research, Sawangi, Wardha (Approval number: DMIHER(DU)/IEC/2025/545 dated: 07/02/2025). Written informed consent will be obtained from patients prior to extraction regarding the use of extracted teeth for research purposes in this study. All extracted teeth will be deidentified before inclusion in the experimental protocol to maintain complete anonymity of the donors. No financial or nonfinancial compensation was provided to the patients for participation in this study or for the use of extracted teeth for research purposes.

## Results

### Overview

Ethical approval was obtained in June 2025. Sample collection of extracted human teeth commenced in July 2025, and 90 extracted teeth have been collected as of January 2026, corresponding to the planned sample size. Laboratory procedures and data analysis have not yet commenced.

### Primary Outcome Measure

The primary outcome measure was the evaluation and comparison of reduction in the CFU count of *E. faecalis* following irrigation by MTR with sonic and laser activation techniques with that of CHX and normal saline.

### Secondary Outcome Measure

The secondary outcome measure was to determine the most effective combination of irrigant and activation technique to target and eliminate *E. faecalis* residing in the root canal space effectively.

## Discussion

Achieving successful root canal outcomes requires the meticulous elimination of all microorganisms harbored within the complicated architecture of the root canal system. Despite advancements in irrigation protocols and antimicrobial strategies, *E. faecalis* remains a major causative agent in treatment failures due to its exceptional resistance and adaptability [1,2]. This facultative anaerobe is known for its ability to penetrate dentinal tubules, survive in nutrient-deprived environments, and resist common intracanal medicaments, including calcium hydroxide [3]. These characteristics underscore the need for not only potent antibacterial agents but also effective activation techniques to enhance their intracanal performance.

In this context, MTR and CHX have emerged as promising irrigants. MTR, a nitroimidazole derivative, is especially effective against anaerobic bacteria and has been used in both systemic and local applications in dental practice [4]. Although its antibacterial spectrum includes *E. faecalis*, the clinical efficacy of MTR may be compromised when used without adjunct activation techniques due to its limited ability to penetrate biofilms [5]. CHX, on the other hand, is a broad-spectrum cationic biguanide that retains its activity in the presence of organic matter and has substantivity, remaining active for prolonged durations postapplication [6]. It has shown superior performance in reducing microbial load within the canal system, especially when used at concentrations above 0.12% [6].

However, the delivery and penetration of these irrigants into canal irregularities are critically dependent on activation techniques. Sonic and laser activation have been shown to improve irrigant flow dynamics, resulting in deeper penetration and enhanced disinfection. Sonic activation systems such as the EndoActivator operate at lower frequencies (1‐10 kHz), creating hydrodynamic agitation that improves smear layer and debris removal. Their greater amplitude allows more effective lateral displacement of irrigants within the root canal [9].

Laser activation, particularly using diode lasers at 940 nm, represents a significant advancement in endodontic disinfection. The unique properties of laser energy—specifically its ability to induce photothermal and photoacoustic effects—result in deeper penetration into dentin and effective bacterial cell wall disruption [10,11]. Unlike conventional irrigants whose efficacy is often limited to the main canal, laser activation facilitates disinfection in otherwise inaccessible microanatomical structures, including lateral canals and apical ramifications [12]. Furthermore, diode lasers are advantageous due to their compact size, cost-effectiveness, and compatibility with clinical settings [11].

The present investigation is designed to explore an under-researched area by directly assessing the antimicrobial performance of MTR, CHX, and saline when activated via laser and sonic methods. While previous studies have highlighted the individual efficacy of these irrigants and activation methods, few have explored their synergistic impact, particularly with MTR as the primary irrigant. Given that metronidazole’s efficacy is enhanced in anaerobic conditions and that *E. faecalis* can adapt to both aerobic and anaerobic environments, activation becomes a pivotal factor in optimizing its bactericidal action [4,5].

Moreover, CHX’s substantivity and ability to disrupt microbial membranes at higher concentrations may complement the mechanical benefits of sonic and laser activation, potentially yielding superior outcomes compared to passive irrigation techniques [6,8]. In contrast, saline serves as a control, allowing for a clear demarcation of the role of activation in enhancing antibacterial performance.

Previous studies support the antibacterial potential of MTR as an endodontic irrigant. Amin et al [16] reported significant bacterial reduction with 5 ml of 0.5% MTR and 2% CHX in primary teeth, with saline showing no effect. Dubey et al [17] found MTR effective against *Bacteroides fragilis* and *Propionibacterium acnes*, while BioPure MTAD showed greater efficacy against *E. faecalis* and *Candida albicans*, highlighting MTR’s limited spectrum. Shweta et al [15] demonstrated that the combination of 0.5% MTR and 2% glutaraldehyde exhibited enhanced antibacterial action over saline, with ultrasonic activation outperforming hand irrigation. These findings justify evaluating the combination of activated MTR and CHX in the present study to enhance efficacy against *E. faecalis*.

The incubation protocol is based on previously validated studies, such as those by Amin et al [16] and Shweta et al [15], which demonstrated reliable biofilm formation of *E. faecalis* following a 7-day incubation period under controlled conditions. These studies used similar in vitro infection models without requiring SEM confirmation to validate biofilm maturity, relying instead on established microbiological protocols and CFU quantification. As this study constitutes Phase I of a multiphase research design, our primary aim is to evaluate the antibacterial efficacy of different irrigants activated by laser and sonic techniques. While SEM is not incorporated in this phase due to resource constraints and focus on microbial reduction outcomes, Phase II is planned to include SEM analysis of selected samples to confirm dentinal tubule penetration and biofilm ultrastructure, thereby augmenting the qualitative depth of findings.

The exploration of enhanced antimicrobial strategies in endodontics, including the use of nanoparticle-modified sealers [18], reflects a broader effort to improve root canal disinfection outcomes. While the present study evaluates the in vitro efficacy of various irrigant–activation combinations, it also underscores the importance of translating such findings into clinical education and practice. As highlighted by Haupt and Kanzow [19], a persistent gap often exists between theoretical understanding and practical competence in endodontics, particularly during preclinical training. Addressing this gap may be critical for the successful clinical application of evidence-based disinfection protocols.

Overall, the findings of this study are anticipated to contribute significantly to clinical endodontics by providing evidence for more effective disinfection protocols. If MTR, when activated, proves comparable or superior to CHX, it may represent a cost-effective and biocompatible alternative for routine endodontic practice. This integrated approach may offer a promising direction toward reducing persistent infections and improving long-term treatment outcomes.

## Supplementary material

10.2196/76783 Multimedia Appendix 1 Sample size formula.

## Abbreviations

BHI: brain heart infusion
CFU: colony-forming units
CHX: chlorhexidine
*E. faecalis*: *Enterococcus faecalis*
MTR: metronidazole
RCT: root canal therapy
