# Supplementary material for: Comparative Evaluation of the Antibacterial Efficacy of Metronidazole, Chlorhexidine, and Normal Saline as Laser- and Sonic-Activated Intracanal Irrigants Against Enterococcus faecalis: In Vitro Study Protocol
Source: JMIR Res Protoc. 2026 Jan 7;15:e76783. doi: 10.2196/76783 (PMC12779102; doi:10.2196/76783)
Supplement: Multimedia Appendix 1 [file resprot-v15-e76783-s001.docx]

Mean CFU count in group II= 50.58

Mean CFU count in group III=27.58

σ1= SD of CFU count in group II=17.33

σ2= SD of CFU count in group II=2.81

For detecting mean difference of 23 i.e. ∆ =50.58-27.58 = 23

K =1

N = (17.33*17.33 + 2.81*2.81)(1.96+0.84)^2^

23*23

=13.59 = 15 patients needed in each group

**Reference :** Jain Shweta et al

**Power of the Test:** 80%

**Level of significance:** 5%( 95% confidence interval)

**Statistical Analysis:**

Statistical analysis will be done by using descriptive and inferential statistics using Chisquare test, Student’s paired, unpaired t test, one way ANOVA, Tukey Test and software used in the analysis will be SPSS 27.0 version and GraphPad Prism 7.0 version and p<0.05 is considered as level of significance.

**Dr. Vijay Y.Babar**

Asst. Prof(Statistics),Community Medicine, JNMC, Sawangi(M), Wardha
